# Preexisting High Expression of Matrix Metalloproteinase-2 in Tunica Media of Saphenous Vein Conduits Is Associated with Unfavorable Long-Term Outcomes after Coronary Artery Bypass Grafting

**DOI:** 10.1155/2013/730721

**Published:** 2013-09-16

**Authors:** Bartlomiej Perek, Agnieszka Malinska, Marcin Misterski, Danuta Ostalska-Nowicka, Maciej Zabel, Anna Perek, Michal Nowicki

**Affiliations:** ^1^Departments of Cardiac Surgery and Transplantology, Poznan University of Medical Sciences, ul. Dluga 1/2, 61-848 Poznan, Poland; ^2^Histology and Embryology, Poznan University of Medical Sciences, ul. Dluga 1/2, 61-848 Poznan, Poland; ^3^Pediatric Cardiology and Nephrology, Poznan University of Medical Sciences, ul. Dluga 1/2, 61-848 Poznan, Poland; ^4^Anesthesiology and Intensive Therapy, Poznan University of Medical Sciences, ul. Dluga 1/2, 61-848 Poznan, Poland

## Abstract

*Introduction*. Migration of the smooth muscle cells (SMCs) to the tunica media in the saphenous vein (SV) transplants is facilitated by matrix metalloproteinases (MMPs). The aim of this study was to identify any associations between expression of MMP-2 or endogenous tissue inhibitors (TIMP-2 and TIMP-3) in the SV segments and late failure of the SV grafts. *Methods*. Two hundred consecutive patients with a mean age of 63.1 ± 8.9 years who underwent primary isolated venous CABG were examined. Patients were retrospectively split into two subgroups, with the SV graft disease (SVGD (+); *n* = 47) or without it (SVGD (−); *n* = 153). In the SV segments, immunohistochemical analysis of the expression of the MMP-2, TIMP-2, and -3 was performed. *Results*. In the SVGD (+) patients, tissue expression of MMP-2 was stronger, whereas that of both TIMPs was weaker than in the SVGD (−) patients. In majority of the SV segments obtained from the SVGD (−) individuals, a balance in MMP and TIMP expressions was found, whereas an upregulation of MMP-2 expression was usually noted in the SVGD (+) subjects. *Conclusion*. The strong expression of MMP-2 accompanied by reduced immunostaining of both TIMPs is associated with the development of the SV graft disease and unfavorable CABG outcomes.

## 1. Introduction

Saphenous vein (SV) is broadly employed in a variety of operations in cardiac, peripheral vascular, general, and even neurosurgery due to its ready availability, ease of obtaining, and generally superior patency to the prosthetic vessels [1–4]. However, the interposition of the SV segments into the arterial system during coronary artery bypass grafting (CABG) initiates a series of biological events within the venous wall [5]. They are thought as adaptation to the high arterial stretch forces and wall shear stresses [6]. In some aspects, they remind the processes typical for arterial remodeling after injury related to angioplasty or stent implantation [7]. On the other hand, these biological processes lead to formation of neointima in the venous grafts. The neointima serves as the foundation for subsequent progressive graft atheroma which eventually results in occlusion of the SV grafts years after CABG [8, 9]. Transformation of the venous smooth muscle cells (vSMCs) from contractile to synthetic and the abnormal proliferation and migration of these cells from the tunica media to the tunica intima through internal elastic lamina are key events in the development of neointima [6, 10]. Before migration of vSMCs, the surrounding extracellular matrix (ECM) must be initially degraded by the matrix metalloproteinases (MMPs) [11, 12]. Of several MMPs, MMP-2 (gelatinase A) possesses a unique ability to degrade elastin and collagen, the main components of the ECM [13]. Under physiological conditions, an excessive proteolytic activity of MMPs is strictly controlled by the endogenous tissue inhibitors of metalloproteinases (TIMPs) [14]. Of four TIMPs, TIMP-2, in addition to inhibiting activity of the MMP, interacts also selectively with the MT1-MMP complex to facilitate the cell-surface activation of pro-MMP-2. TIMP-2 may also inhibit the development and destabilization of the atherosclerotic plaques [15]. TIMP-3, the only TIMP that is incorporated into the ECM, has very broad inhibition spectrum, including not only all MMPs but also several disintegrin metalloproteinases (ADAM and ADAMTS), and also manifests a potent antiapoptotic activity [16]. An appropriate balance between MMPs and TIMPs is considered to be of paramount importance to preserve normal structure and function of the vessels. Thus, in many vascular diseases, such as varicose veins, aortic aneurysms, or hypertension, an existing imbalance in activity of the MMPs versus TIMPs may be underlying pathology [13, 14, 17].

The late CABG outcomes with respect to control of the coronary artery disease (CAD) symptoms are determined not only by the progression of atherosclerosis in the native coronary arteries, but also by patency of the conduits used for the CABG procedures [18]. Although arterial grafts, such as internal thoracic arteries, have a higher late patency rate than veins, the SV autologous conduits are still widely used as the aortocoronary bypass grafts [19, 20].

Thus, the aim of this study was to identify any associations between expression of MMP-2 or the endogenous tissue inhibitors (TIMP-2 and TIMP-3) in the tunica media of the SV transplants and the development of the late graft disease that impact negatively on the late CABG outcomes.

## 2. Patients and Methods

### 2.1. Study Group

The Local Ethics Committee has approved the study protocol (no. 1201/08), and each study participant gave informed written consent.

Two hundred consecutive patients (148 males (74.0%) and 52 females (26.0%)) with the mean age of 63.1 ± 8.9 years (44–85 years) who underwent primary isolated nonemergent CABG with at least one venous aortocoronary bypass graft between September 2008 and March 2009 were enrolled in the prospective observational study. They were retrospectively split into two subgroups according to the findings of the last follow-up coronary angiography, with the SV graft disease (SVGD (+) patients; *n* = 47) or without it (SVGD (−) patients; *n* = 153). The exclusion criteria included emergent priority of surgery, severe chronic ischemia of the peripheral arteries (*arterioselerosis obliterans*), and any lower extremity vein pathologies. All patients enrolled in the study but died either during in-hospital stay or the follow-up period were not included due to a lack of availability of the follow-up coronary angiography. Additionally, all individuals who developed myocardial infarction during the first month following CABG procedure were also excluded from the study. The early occlusion of the SV grafts, defined as failure up to 30 days after operation, has been attributed to thrombosis or to improper surgical management rather than to the formation of neointima with subsequent atheroma [9]. Selected preoperative data of both subgroups are summarized in Table 1.

### 2.2. Tissue Samples Collection

SVs were obtained routinely through full-length thigh incisions over the entire course of the vein. Surgeons paid special attention to meticulous surgical technique. Key points included minimal manipulations with the grafts (“no-touch” technique) and prevention of extensive dilation of the conduits. The venous segments with macroscopic signs of both the preexisted abnormalities (thick wall, pathological dilation, inflammation of the adjacent tissues, and severe adhesions around the vein) and intraoperative injury were disqualified. In all cases, the most distal segments of the harvested SV conduits at least 2.0 cm long were taken for histological examinations.

### 2.3. Immunohistochemistry for MMP and TIMPs in the SV Conduits

The SV segments were carefully rinsed with 0.9% NaCl at the room temperature, slightly dilated, and fixed in Bouin's solution for 2 to 3 hours. They were dehydrated and then embedded in paraffin blocks using a routine procedure. Eventually, they were cut into the 5 *μ*m sections on a semiautomatic rotary microtome (Leica RM 2145, Leica Microsystems, Nussloch, Germany). 

All immunohistochemical analyses employed the Dako REAL EnVision Detection System, Peroxidase/DAB, Rabbit/Mouse, K5007 (Dako, Copenhagen, Denmark). After deparaffinization with xylene and gradual rehydratation, the endogenous peroxidase activity was blocked with 10% hydrogen peroxide (v/v) treatment for 10 minutes. Then, the sections were incubated with the following mouse monoclonal antibodies: anti-MMP-2 (dilution 1 : 50, MAB13431, Millipore, Darmstadt, Germany), anti-TIMP-2 (1 : 200, MAB3310, Millipore), and anti-TIMP-3 (dilution 1 : 100, MAB3318, Millipore) for 18 h at 4°C and then for 60 min at room temperature. The samples were rinsed and then incubated for 60 min at room temperature with a dextran coupled with peroxidase molecules and goat secondary antibody molecules against mouse immunoglobulins (30 min at room temperature). The peroxidase reaction was developed using diaminobenzidine.

The intensity of cytoplasmic expression of the proteins was assessed using the semiquantitative IRS (immunoreactive score) scale according to Remmele and Stegner [22]. It takes into account the percentage of positive cells (scale from 0 to 4) and the intensity of the color reaction (scale from 0 to 3), and final score that ranges from 0 to 12 is a product of points given for individual traits (Table 2). On the base of IRS, the expression of the cytoplasmic proteins was defined as negative (IRS 0-1), positive weak (IRS 2-3), positive moderate (IRS 4–6), or strong (IRS 8–12). All tissue sections were analyzed using an AxioImager Z.1 light microscope, and representative images were captured with an attached AxioCam MRc5 digital camera (Carl Zeiss MicroImaging GmbH, Göttingen, Germany).

For uniformity, immunohistochemical analyses of the expression of proteins in each vessel section were done within 10 representative microscopic fields (200x magnification). The expression of the proteins was evaluated blindly and independently by two pathologists on the coded samples that included also both negative and positive controls. A final score of the expression of a given protein in each SV sample, that was entered in statistical analysis, was the median score derived from 20 assessments (2 pathologists × 10 microscopic fields). The negative controls consisted of the sections incubated with nonimmune IgG1 (X0931, Dako) and the sections in which the primary or secondary antibody was omitted. All the sections corresponding to a vein sample of an individual patient were processed in the same immunohistochemical experiment. In addition, the serial sections were stained in the subsequent experiments as positive controls to determine the consistency of staining.

### 2.4. Clinical Outcomes and Late Patency of the SV Grafts

All patients were followed up systematically in the outpatient facilities. Unless contraindicated, the patients were routinely treated medically with statins and acetylsalicylic acid/clopidogrel. All CABG subjects were also educated on how to control risk factors for the progression of CAD. At the end of followup that lasted 50.5 ± 9.1 months and was completed by 202 patients (96.2%) patients, clinical and angiographic assessments were carried out. Follow-up coronary status was evaluated according to Canadian Cardiovascular Society (CCS) classification. Additionally, at the end of follow-up period, all aortocoronary bypass grafts were examined by the coronary angiography (*n* = 186) or multidetector spiral computed tomography angiography (MSCT) (*n* = 24). MSCT was offered only to subjects who withdrew consent for the follow-up invasive coronary examination. However, they would have agreed to undergo coronary angiography if MSCT had revealed significant lesions. SVGD, a primary angiographic endpoint, was defined as graft stenosis that exceeded 70%.

#### 2.4.1. Data Management and Statistical Analysis

All continuous variables were estimated for normality with the Shapiro-Wilk *W* test. When the values were normally distributed, they were expressed as the means ± standard deviations and then compared using Student's *t*-test. Ordinal variables (i.e., points in IRS scale) were expressed as the median with the 25th and 75th percentiles (for IRS scale comparison) and were analyzed with the Mann-Whitney *U* test. Nominal data were presented as the number (*n*) and percentages (%), and the differences between the examined groups were analyzed using single chi-square tests. A *P* value <0.05 was considered statistically significant. All statistical analyses were performed with Statistica 9.0 for Windows (StatSoft, Inc., Tulsa, OK, USA).

## 3. Results

### 3.1. Late CABG Outcomes

In the last follow-up examination, 47 patients reached angiographic study endpoint. In two of them, MSCT revealed occluded SV grafts, and these findings were confirmed in the coronary angiography. During the postdischarge follow-up period, 11 patients developed acute coronary syndromes (ACSs) (non-ST segment elevation myocardial infarction (NSTEMI) (*n* = 5), ST segment elevation myocardial infarction (STEMI) (*n* = 4), and unstable angina (*n* = 2)). Thus, in these individuals, coronary angiography was carried out earlier (averaged 15.2 ± 9.3 months after surgery) than at the end of follow-up period. The follow-up coronary angiography revealed also failure of the grafts in 31 study participants who manifested an exacerbation of angina and in 5 subjects without any symptoms of the progression of CAD. At the time of the last follow-up examination, all of the latter subgroup had suffered from diabetes treated with insulin for at least 5 years.

The progression of atherosclerosis in the native coronary arteries (defined if the culprit lesions reducing vessel diameter by at least 70% were located distally to anastomoses with the grafts or in the nonbypassed coronary arteries) was seen in 15 patients, including 4 cases without significant changes in the grafts. However, no complete occlusions in the bypassed native coronary arteries distally to the anastomoses sites were visualized in the follow-up coronary angiography. Detailed locations of the significant lesions are summarized in Table 2. Thus, in majority of patients with the clinical progression of CAD (*n* = 38, 90.5%), failure of the SV grafts (isolated or concomitant with the progression of CAD in the arterial grafts or the native coronary arteries) was diagnosed.

### 3.2. Tissue Expression of MMP and TIMPs

In majority of the SV samples, no or weak expression of either MMP-2 (no expression in 54 (27.0%), positive weak in 60 (30.0%) cases) or TIMP-3 (no expression in 77 (38.5%) and weak positive in 49 (29.5%) cases) was noted, whereas strong immunostaining was detected in approximately 10% (both in 21 samples). Generally, tissue expression of TIMP-2 was more pronounced than MMP-2 or TIMP-3, and in approximately two-fold more samples (39 (19.5%)), strong immunostaining of TIMP-2 was noted. A balanced tissue expression of MMP-2 versus TIMP-2 was found in 123 (61.5%) while MMP-2 versus TIMP-3 in 129 (64.5%) SV sections.

**Figure 1:**
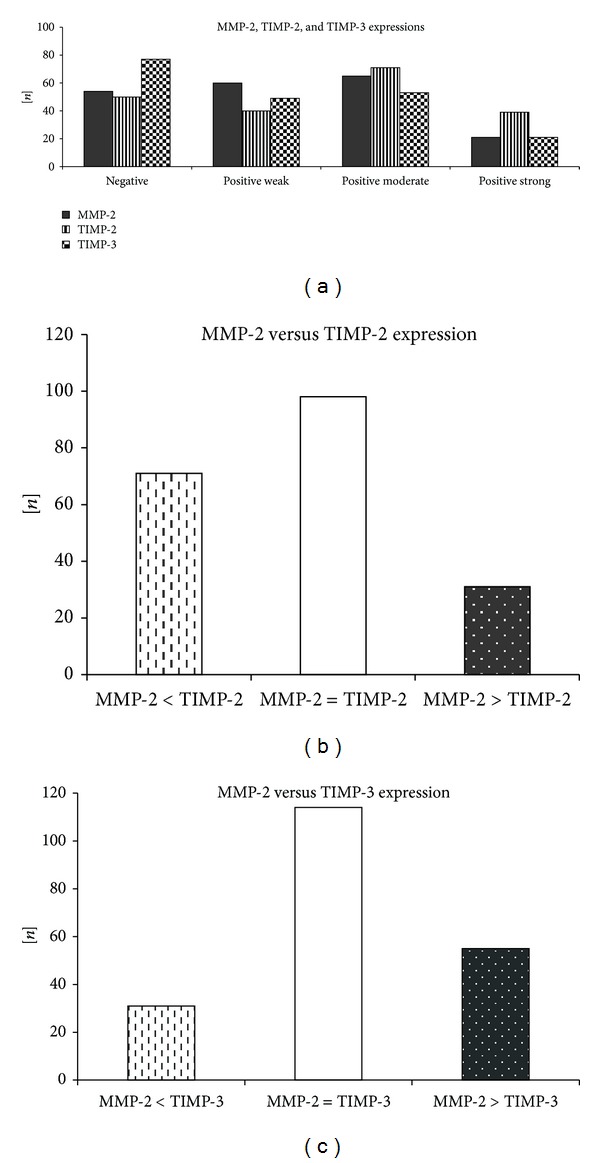
Detailed expressions of MMP-2, TIMP-2, and TIMP-3 in the autologous SV segments.

The detailed expressions of both MMP-2 and TIMPs are presented on Figure 1.

### 3.3. SVGD (+) versus SVGD (−) Patients

Generally, the SVGD (+) patients were significantly younger, more often were active smokers, and manifested the higher prevalence of renal failure (defined as glomerular filtration rate (GFR) below 50 mL/min/1.73 m^2^) and diabetes mellitus treated with insulin than the SVGD (−) patients. More detailed data regarding risk factors for the development of atherosclerosis are outlined in Table 1.

### 3.4. Tissue Expression of MMP or TIMPs and SVGD

**Figure 2:**
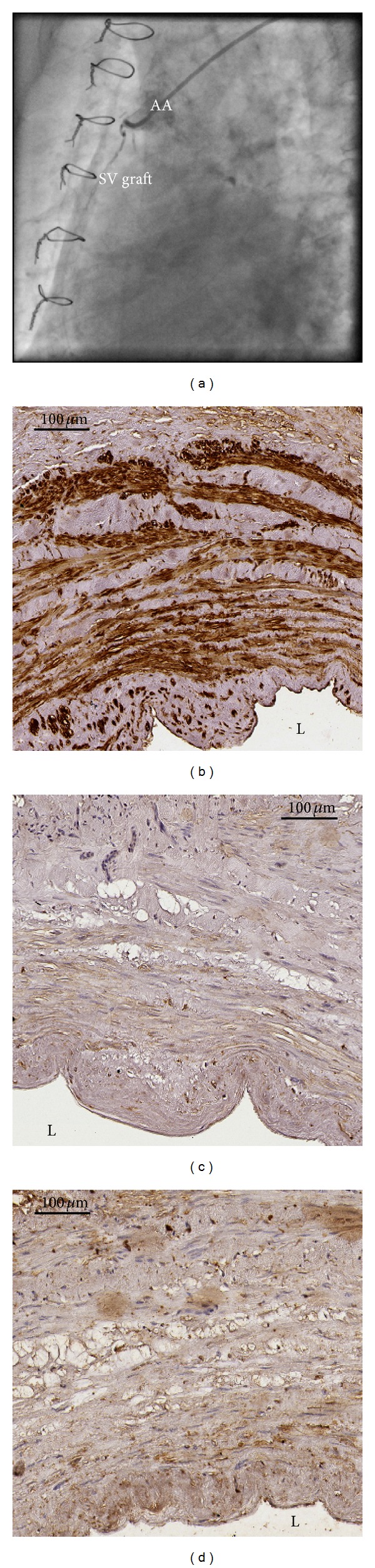
Occluded SV graft to the right coronary artery was found in the follow up coronary angiography (Figure 2(a)) that was perfomed in a patients with clinical progression of CAD. Immunohistochemically assessed expression of MMP-2 (Figure 2(b)) was stronger (IRS scale 9) than either TIMP-2 (Figure 2(c)) (IRS scale 1) or TIMP-3 (Figure 2(d)) (IRS scale 1). Abbreviations: AA = ascending aorta, L = lumen of the graft, and SV = saphenous vein.

**Figure 3:**
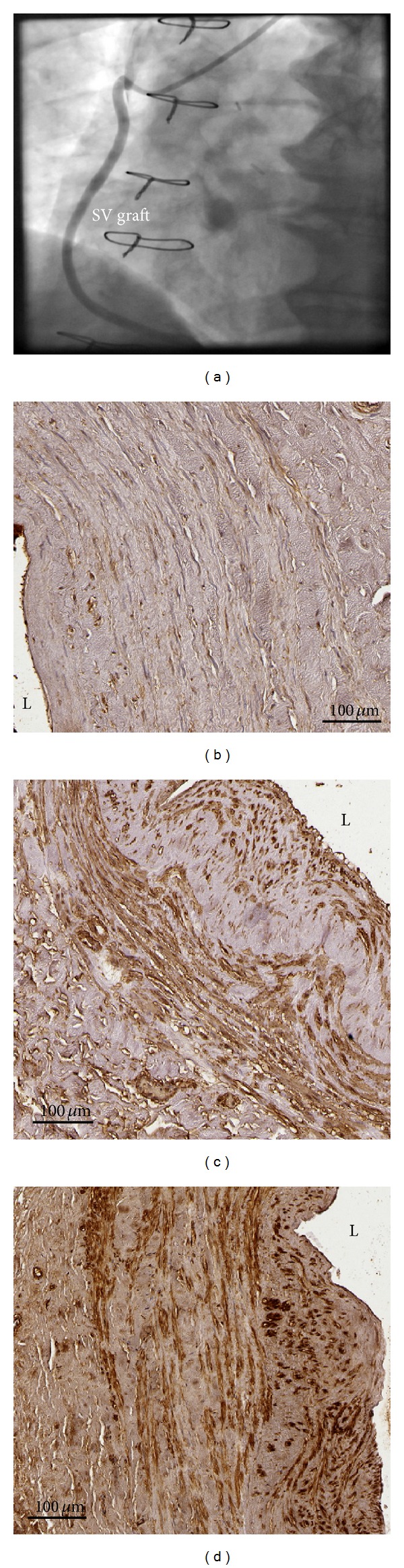
The last follow-up coronary angiography (Figure 3(a)) in the SVGD (−) patient revealed the SV graft without any changes. In immunohistochemistry, a tissue expression of MMP-2 (Figure 3(b)) was markedly weaker (IRS scale 1) than either TIMP-2 (Figure 3(c)) (IRS scale 8) or TIMP-3 (Figure 3(d)) (IRS scale 6). Abbreviations: L = lumen of the graft, SV = saphenous vein.

The expression of either MMP-2 or its inhibitors, such as TIMP-2 and TIMP-3, differed significantly between the SVGD (−) and SVGD (+) patients (Tables 3 and 4). In the latter group, tissue expression of MMP-2 presented as IRS score was significantly higher, whereas both TIMPs scored markedly weaker than in the SVGD (−) patients. In majority of the SV segments of the SVGD (−) individuals, a balanced expression of MMP versus TIMP was found, whereas in the SVGD (+) subjects, an upregulation of MMP-2 versus TIMPs expression was usually noted (Table 4, Figures 2 and 3). Additionally, in 12 cases of the SVGD (−) patients, positive strong expression of MMP-2 was accompanied by also positive strong expression of both TIMP-2 and TIMP-3.

## 4. Discussion

This study showed that the postdischarge clinical deterioration of CAD in majority of cases resulted from the SV grafts failure rather than the progression of atherosclerosis in the native coronary arteries. Only in 3 cases (6.4%) who reached angiographic study endpoint, the SV graft failure was accompanied by significant progression of CAD in the recipient native coronary arteries. We must stress again that no complete occlusions of the bypassed arteries were visualized. Thus, SV graft disease is probably not related directly to the formation of the significant atherosclerotic plaques in the distal segments of the bypassed coronary arteries. However, prolongation of the follow-up period for 10 to 15 years may change the significance of the morphological pathologies in the grafts and in the native coronary vascular bed. Because of a fact that the SVs are still the most common grafts used in the CABG subjects, large and multicenter clinical trials that would involve more patients should be carried out to identify the subgroups of individuals who would benefit the most from venous aortocoronary grafts in the terms of optimal postdischarge control of the CAD symptoms. In the previous study published by our team, we showed that some histological ageing-related variations, such as progressive hypotrophy of SV wall and the tunica media, might have positive impact on the long-term CABG followup [23]. A comparison of the SVGD (+) to SVGD (−) demographic data revealed that a population with preterm failure of the SV grafts was significantly younger. We also found a higher prevalence and longer duration of the insulin-treated diabetes and active smokers among the SVGD (+) patients. Thus, in our daily clinical practice in cardiac surgery we should probably avoid using the SV grafts in these subsets of CABG subjects. Our results support the earlier opinions that the most appropriate grafts for the diabetics are arterial conduits [24, 25].

The key histological finding in this study was that the preexisting tissue expression of MMP-2 in the SV wall of normal macroscopic appearance was not uniform, and this variability could be at least associated with the long-term CABG outcomes. In the SVGD (+) patients, MMP-2 expression evaluated as either positive strong or positive moderate according to IRS scoring system was noted significantly more often than in the SVGD (−) subjects. However, this is not a novel finding. The elevated expression of the MMPs, particularly MMP-2 and MMP-9 has been identified as one of the crucial events in the processes of the vascular remodeling [26]. These MMPs were demonstrated in the experimental animal models to be mandatory molecules to promote proliferation and migration of the vSMCs from the tunica media to the tunica intima. Previously published study dealing with smoking as a risk factor for accelerated failure of the SV grafts disclosed that upregulated expression of MMP-2 gene in the SV transplants obtained from the heavy smokers might increase the prevalence of vein graft disease within the long-term follow-up period [27]. Overexpression of MMP-2 was also found in the animals models of the SV segments into the carotid artery interposition [28].

We showed in this study that not only absolute expression of the selected proteins but also an imbalance between tissue activities of MMP-2 and TIMPs in the SV wall might have an adverse impact on the late fate of the SV grafts. Tissue remodeling that involves MMPs and TIMPs is considered as a normal dynamic biological process [29]. This fine harmony is dysregulated in several vascular pathologies of both arteries and veins [14, 18]. For example, the higher expression of MMPs was found in the abdominal aortic aneurysms, gestational hypertension, and arteries after intravascular interventions [30, 31]. On the other hand, the stronger expression of TIMPs was shown to facilitate deposition of the connective tissue in the venous wall that resulted in reduced tissue turnover and in thicker vein wall [17]. We found for the first time that preexisting at the time of surgery stronger expression of MMP-2 than of TIMPs was linked to the development of SVDG during the follow-up period after CABG. This phenomenon was found in majority of the SVGD (+) patients whereas in less than 5% of the SVDG (−) cases. Interestingly, in all cases of the SVGD (−), positive strong expression of MMP-2 was balanced by pronounced tissue activities of both TIMP-2 and TIMP-3. Thus, our study proved that intrinsic ability of the SV wall to preserve tissue homeostasis of the proteolytic proteins was at least as important as absolute expression of the particular MMPs and TIMPs.

Another issue of importance is to find any explanation for why in some SV segments with normal macroscopic appearance the activity of MMP-2 is upregulated. It was reported previously that MMP-2 was produced and released from the vascular SMCs after stimulation by a variety of the local substances strictly related to the clinical risk factors for the development of atherosclerosis, such as diabetes mellitus, hypertension, smoking, and ageing [14, 32, 33]. On the base of the cigarette smokers, it was found that even the quitters some time prior to surgery still manifested an increased activity of the MMP-2 gene [27]. Thus, it may be possible that cellular and tissue effects of the aforementioned factors promoting atherosclerosis could be permanent or not completely reversible. We found significant differences between the groups (SVDG (−) versus SVGD (+)) with respect to the active smokers rate, the prevalence, and duration of diabetes treated with insulin.

Majority of the publications dealing with the associations between the clinical variables and the expression of MMPs were carried out on arteries. In the arteries, ageing was found to be usually related to the development of wall hypertrophy and formation of the connective tissue deposits. Contrary to the arteries, in this study on the SV grafts, we revealed that the younger age of the SV transplant recipients at the time of surgery may be linked to the adverse long-term outcomes. One of the possible explanations may be the findings of the earlier study of our team [23]. We showed that ageing-related progressive thinning of the SV wall resulted from hypotrophy of the tunica media and degeneration of the SMCs.

We are aware of some study limitations. First of all, nowadays, it is not possible to reveal definitely the predominant cause of the SV graft failure in the coronary angiography, a routine method to check the quality and function of the grafts. We can just visualize the final results after a given period of time. However, based on the experimental studies on the animal models, we may only presume that the main pathway leads through the formation of neointima to subsequent development of the occluding atherosclerosis. Moreover, the immunohistochemical assessment of the protein expressions, both active and bound forms, was detected. Thus, it is possible in some samples that real expression of MMPs or/and TIMPs could be overestimated. Additionally, by virtue of a relatively small number of the participants who reached angiographic study endpoint, we were not justified to perform multivariate analysis to find the independent risk factors for the development of SVDG. However, we plan to continue our study that will involve more patients with a significantly prolonged follow-up period.

## 5. Conclusion

Dysregulation of MMP-2 and TIMPs expression in the SV segments may have a potential impact on the prevalence of the SV graft failure during the long-term follow-up period. A strong tissue expression of MMP-2 accompanied by reduced TIMPs immunostaining is associated with the development of the SV graft disease and unfavorable long-term CABG outcomes.

## Figures and Tables

**Table 1:** Selected preoperative demographic and clinical data.

Variable	SVGD (+) *N* = 47	SVGD (−) *N* = 153	*P* value
Age [years]	57.1 ± 7.5	64.7 ± 8.7	**<0.001**
Gender (M/F)	37/10	111/42	0.383
Obesity^a^	19 (40.4)	68 (44.4)	0.875
Arterial hypertension	37 (78.7)	105 (68.6)	0.089
Diabetes	20 (42.6)	69 (45.1)	0.675
Diabetes treated with insulin	12 (25.5)	19 (12.4)	**0.022**
Therapy duration [months]	76.8 ± 10.6	20.4 ± 3.6	**0.013**
Hyperlipidemia	21 (44.7)	68 (44.4)	0.967
Renal failure^b^	7 (14.9)	7 (4.6)	0.042
Active smoking^c^	13 (27.7)	18 (11.8)	**0.008**

Continuous variables are expressed as the mean ± standard deviation; nominal data as the number (*n*) with percentages (%).

^a^If body mass index (BMI) exceeded 30 kg/m^2^; ^b^glomerular filtration rate (GFR) based on creatinine clearance calculated using Cockcroft-Gault formula [21] was below 50 mL/min/1.73 m^2^; ^c^defined as currently smoking one cigarette a day or quit smoking less than one month prior to surgery.

Abbreviation: SVGD: saphenous vein graft disease.

**Table 2:** Detailed location of the significant lesions in the follow-up coronary angiography.

Location	Patients [*n*]
Grafts without native arteries	36 (34 SV grafts only, 2 SV grafts + LIMA)
SV grafts + nonbypassed coronary arteries	8
SV grafts + bypassed coronary arteries	3^a^
Native coronary arteries without grafts	4^b^

^a^No complete occlusions in the bypassed native coronary arteries; ^b^only in the nonbypassed native coronary arteries.

Abbreviations: LIMA: left internal mammary artery, SV: saphenous vein.

**Table 3:** Mean tissue expressions of MMP-2, TIMP-2, and TIMP-3 in the SV segments obtained from patients who developed the SV graft disease (SVGD (+)) versus individuals without SV graft failure during the follow-up period (SVGD (−)).

IRS scale	SVGD (+) *N* = 47	SVGD (−) *N* = 153	*P* value
MMP-2	4 (2, 6)	3 (1, 4)	**0.001**
TIMP-2	2 (0, 6)	4 (1, 6)	**0.044**
TIMP-3	0 (0, 4)	2 (0, 4)	**0.015**
Ratio MMP-2 : TIMP-2	1.7 (1, 2.5)	0.2 (0, 1)	**0.002**
Ratio MMP-2 : TIMP-3	1.9 (1, 3)	0.4 (0, 1)	**0.002**

IRS scale is expressed as the median with the 25th and 75th percentiles; categorical variables as the number (*n*) with percentages (%).

Abbreviations: IRS: immunoreactive score, MMP: metalloproteinase, TIMP: tissue inhibitor of metalloproteinase, and SVGD: saphenous vein graft disease.

**Table 4:** Distribution of negative and positive expressions of MMP-2, TIMP-2, and TIMP-3 in the SV segments obtained from patients who developed the SV graft disease (SVGD (+)) versus individuals without SV graft failure during the follow-up period (SVGD (−)).

Variable	SVGD (+) *N* = 47	SVGD (−) *N* = 153	*P* value
MMP-2			
Negative	10 (21.3)	44 (28.8)	0.312
Positive weak	6 (12.8)	54 (35.3)	**0.003**
Positive moderate	22 (46.8)	43 (28.1)	**0.017**
Positive strong	9 (19.1)	12 (7.8)	**0.027**
TIMP-2			
Negative	18 (38.3)	32 (20.9)	**0.016**
Positive weak	10 (21.3)	30 (19.6)	0.802
Positive moderate	13 (27.6)	58 (37.9)	0.199
Positive strong	6 (12.8)	33 (21.6)	0.182
TIMP-3			
Negative	26 (55.4)	51 (33.3)	**0.007**
Positive weak	9 (19.1)	40 (26.1)	0.329
Positive moderate	9 (19.1)	44 (28.8)	0.191
Positive strong	3 (6.4)	18 (11.8)	0.293

Balance in expression of MMP-2 versus TIMPs			

MMP-2 versus TIMP-2			
MMP-2 < TIMP-2	2 (4.3)	47 (30.7)	**<0.001**
MMP-2 = TIMP-2	19 (40.4)	104 (68.0)	**<0.001**
MMP-2 > TIMP-2	26 (55.3)	2 (1.3)	**<0.001**
MMP-2 versus TIMP-3			
MMP-2 < TIMP-3	2 (4.3)	32 (20.9)	**0.008**
MMP-2 = TIMP-3	14 (29.8)	115 (75.2)	**<0.001**
MMP-2 > TIMP-3	31 (65.9)	6 (3.9)	**<0.001**

*Variables are presented as the number (*n*) with percentages (%).

Abbreviations: IRS: immunoreactive score, MMP: metalloproteinase, TIMP: tissue inhibitor of metalloproteinase, and SVGD: saphenous vein graft disease.
